# Seizure Activity Occurs in the Collagenase but not the Blood Infusion Model of Striatal Hemorrhagic Stroke in Rats

**DOI:** 10.1007/s12975-014-0361-y

**Published:** 2014-07-24

**Authors:** Ana C. Klahr, Clayton T. Dickson, Frederick Colbourne

**Affiliations:** 1 Centre for Neuroscience, University of Alberta, P217 BSB, Edmonton, AB T6G 2E9 Canada; 2 Department of Psychology, University of Alberta, P217 BSB, Edmonton, AB T6G 2E9 Canada

**Keywords:** Intracerebral hemorrhage, Stroke, Collagenase, Autologous blood, Seizures, EEG

## Abstract

Seizures are a frequent complication of brain injury, including intracerebral hemorrhage (ICH), where seizures occur in about a third of patients. Rodents are used to study pathophysiology and neuroprotective therapies after ICH, but there have been no studies assessing the occurrence of seizures in these models. Thus, we compared seizure incidence and characteristics after infusing collagenase (0.14 U), which degrades blood vessels, and autologous blood (100 μL) into the striatum of rats. Saline was infused in others as a negative control, whereas iron, a by-product of degrading erythrocytes, served as a positive control. Ipsilateral and contralateral electroencephalographic (EEG) activity was continuously monitored with telemetry probes for a week after the stroke. There were no electrographic abnormalities during baseline recordings. As expected, saline did not elicit any epileptiform activity whereas iron caused seizure activity. Seizures occurred in 66 % of the collagenase group between 10 and 36 h, their duration ranged from 5 to 90 s, and these events were mostly observed bilaterally. No such activity occurred after blood infusion despite comparable lesion sizes of 32.5 and 40.9 mm^3^ in the collagenase and blood models, respectively (*p* = 0.222). Therefore, seizures are a common acute occurrence in the collagenase but not whole blood models of striatal ICH (*p* = 0.028, for incidence). These findings have potential implications for ICH studies such as for understanding model differences, helping select which model to use, and determining how seizures may affect or be affected by treatments applied after stroke.

## Introduction

Intracerebral hemorrhage (ICH) occurs in ~15 % of stroke patients, leading to ~50 % mortality and significant disability in survivors [1, 2]. So far, there are no specific neuroprotective therapies for ICH, although survivors benefit from rehabilitation. Thus, it is important to fully understand those factors that affect outcome after ICH in order to improve medical management and further limit death and disability. For instance, seizures are a common occurrence after ICH or even a presenting sign of an ICH. About 4–20 % of ICH patients will suffer from clinical seizures (e.g., convulsions), whereas 30 % of ICH victims will have subclinical seizures observable on an electroencephalogram (EEG; [2–4]). Current data suggests that the risk of seizures occurring within the first month is 8 % [3], and the risk of a seizure occurring after the first month and within the first year is 3 % [4]. Still, this could be an underestimate caused by the lack of continuous EEG monitoring in patients.

Intuitively, seizures are expected to worsen outcome after an ICH. Seizures can exacerbate excitotoxicity and oxidative stress [5, 6], augment metabolic rate [7], and cause re-bleeding or increased bleeding due to elevated blood pressure and blood flow during seizures [3, 8, 7]. Intracranial pressure (ICP) also rises due to seizures [9], which can cause complications after ICH (e.g., herniation) and increase mortality [2]. Seizures may also cause aberrant brain plasticity (e.g., larger cortical maps) and impair recovery [10]. Lastly, even though the number of ICH patients that develop epilepsy is relatively low (2–5 %), the incidence of one seizure increases the chance of developing epilepsy [2, 4, 3].

All of this illustrates why seizures could be harmful. Clinical studies on this topic, however, have not consistently found that seizures are detrimental [11, 2–4], although some support this notion [12–14]. This variability among clinical studies could be attributed to several factors, such as inclusion criteria, methods for measuring EEG, lack of continuous EEG monitoring, use of anti-epileptic drugs (AEDs), among others. Current guidelines suggest that ICH patients with a depressed mental state should have continuous EEG monitoring, as most seizure activity occurring after ICH is subclinical [2–4, 15]. Any seizure activity ought to be treated intravenously with an AED, and if seizure activity persists, AED treatment may continue orally. Prophylactic administration of AEDs, however, has been discouraged after evidence from studies indicating a worsening of outcome caused by administration of phenytoin before any signs of seizure activity after not only ICH [15, 16] but also traumatic brain injury [17].

The incidence and consequences of seizures after an ICH have not been well studied in animal models. Thus far, swine studies have shown that excitability increases in certain areas of the brain after ICH [18]. Others have shown that in rodents, the most widely used ICH model, intracerebral infusions of blood components such as thrombin [19] and iron [20] cause seizures. To our knowledge, there have been no formal evaluations of seizure activity in the common rodent models of ICH, which involve injecting autologous blood [21] or collagenase [22] into the brain. Unlike the whole blood injection, bacterial collagenase, an enzyme that breaks down the basal lamina, causes bleeding over hours mimicking what frequently occurs in ICH patients [22, 23]. Often, investigators target the striatum as it is a common site of ICH in humans and because it can contain a large hematoma that results in persistent, easily quantified, behavioral impairments [24]. In this study, we induced a moderate-sized striatal ICH in rats by injecting collagenase or whole blood, and we monitored rats with an implanted EEG telemetry probe for a week after the stroke. By using telemetry, we were able to continuously record EEG in freely moving untethered animals, which is the least stressful method for these animals. The objective of this study was to determine the incidence and characteristics of seizures that occur in these animal models of ICH.

## Methods

### Subjects

Twenty male Sprague-Dawley rats (250–400 g, ~3 months old) obtained from the Biosciences breeding colony at the University of Alberta were assigned to either the collagenase, whole blood, or saline group. As a positive control, a rat received an injection of FeCl_2_. Food (Purina rodent chow) and water were provided ad lib and rats were housed individually in a temperature- and humidity-controlled room (lights on from 7 a.m.–7 p.m.).

### EEG Probe Implantation

**Fig.1:**
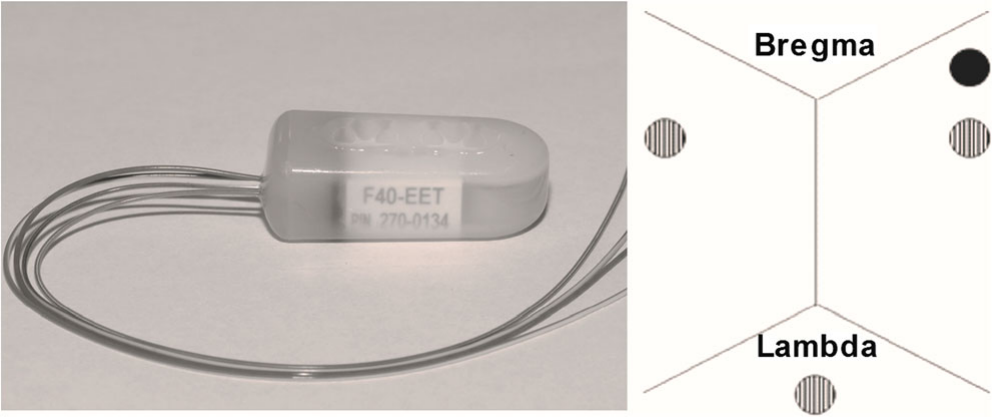
Telemetry probe inserted either in the peritoneum or under the skin of the neck (*left*). Leads were attached to screws (*striped circle*) on the skull and cemented. One channel recorded from the ipsilateral hemisphere next to the site of injection (*full circle*) and the other channel recorded from the contralateral side. Negative leads were connected to a screw posterior to Lambda. No further grounding was required

Surgical procedures were performed aseptically. Rats were anesthetized with isoflurane (4 % induction, 1.5–2.5 % maintenance in 60 % N_2_O, balance O_2_) and body temperature was maintained at 37 °C during anesthesia with a heated water blanket and a rectal temperature probe. An EEG telemetry probe (F40EET, Data Sciences International, St. Paul, MN) was inserted either in the peritoneal cavity or the neck (dorsal S.C. placement), and the leads were channeled under the skin and attached to screws stereotaxically placed ipsilateral (AP −1.5, ML 4) and contralateral (AP −1.5, ML −4) to the injection site (see Fig. 1). The leads were secured to the screws (0–80 × 3–32; Plastics One, Roanoke, VA) with dental cement. These telemetry probes measure EEG (sampled at 500 Hz, low-pass filtered at 100 Hz) and temperature and movement activity. The latter is detected by changes in signal strength as the probe moves across the receiver (Data Sciences International), providing a relative measure of activity [25].

### Iron Injection

As a positive control to assess the ability of the EEG probe to detect seizures, we injected iron in the rat striatum (*N* = 1). Immediately following attachment of the electrical leads, a hole was drilled (AP 0.5, ML 3.5) and a Hamilton 26-gauge needle was inserted 6.5 mm into the striatum to infuse 38.0 μg of FeCl_2_ in 30 μL of saline [26, 27]. The injection was completed over 10 min and the needle was removed after an additional 10 min. Clips were used to close the wound. The rat was euthanized 1 week later.

### Collagenase, Blood, and Saline Injections

For the saline (*N* = 3) and five of the collagenase rats (*N* = 10), a baseline recording period of 1 week was undertaken before the injection. No baseline recording was done for the whole blood rats (*N* = 6). Following baseline recordings, or directly after the securing of electrical leads, a hole was drilled (AP 0.5, ML 3.5) and a Hamilton 26-gauge needle was inserted 6.5 mm into the striatum. Either saline, 100 μL of autologous whole blood (from tail artery), or 0.14 U of bacterial collagenase in 0.7 μL of sterile saline was infused over 5 or 10 min (blood model) and the needle was removed after an additional 5 or 10 min [23]. Clips were used to close the wound. Saline control and whole blood rats were euthanized after 7 days whereas the collagenase rats were euthanized between 11 and 66 days. This difference in survival times was due to differences in probe placement and technical difficulties that ensued. For instance, probe placements in the neck region caused irritation due to collection of fluid (seroma).

### Euthanasia and Lesion Volume Assessment

Rats were injected with sodium pentobarbital (100 mg/kg, i.p.) and then perfused with 0.9 % saline followed by 10 % neutral buffered formalin. Brains were extracted, cryostat-sectioned at 40 μm, and stained with cresyl violet. Coronal sections taken every 200 μm were then analyzed with Image J [28], as routinely done on digitized images extending anterior, through, and posterior to the lesion. The volume of each hemisphere was calculated as follows: (average area of complete coronal section of the hemisphere − area of damage − ventricle) × interval between sections × number of sections [26, 23]. This method takes into account both areas of injury as well as atrophy and ventricular dilation.

### EEG Analysis

Baseline and post-infusion EEG traces were visualized with Dataquest A.R.T. 2.3 system (Data Sciences International) and the incidence, duration of seizures, as well as the time to seizure onset from injection were recorded. In three rats, an extended period of 30 days was further analyzed in this way. We compared 5-min epochs of non-epileptiform activity from the day prior to the injection with recordings taken at least 3 days post-stroke from the rats that had a 1-week baseline recording (collagenase *N* = 4; saline *N* = 3) in order to detect any changes in otherwise normal-looking EEG. We did not find a significant difference between the day prior to collagenase infusion and day 3 after stroke. Therefore, we considered traces from the third day post-collagenase infusion to be an additional substitute baseline measure for all collagenase rats. Baseline and putative epileptiform traces were exported and analyzed using custom code written in MATLAB (R2012a, Mathworks, Natick, MA). We computed for each signal the root mean square (RMS), the power spectral density using Welch’s averaged modified periodogram method (6-s window, 2-s overlap), as well as dual channel coherence for ipsilateral and contralateral recordings (3-s window; 1-s overlap). Field signals and spectra were plotted for comparison with baseline measures using Origin 9.1 (Microcal Software, Northampton, MA). We compared all putative epileptiform measures, including RMS and 95 % confidence intervals of amplitude fluctuations, to those taken during baseline/control conditions in order to confirm that the events were abnormal. For ictal traces longer than 25 s, power spectra were compared to those baseline traces equal in duration to determine total increases in power, frequencies significantly affected by the seizures, as well as bilateral coherence. A randomized coherence distribution based on a series of sequential time-shifted (and time-reversed) coherence computations from these actual traces was computed to calculate the coherence significance level. The maximum 95 % confidence limit for this randomized distribution (throughout the frequency range) across multiple datasets having durations ranging from 27 to 87 s ranged from 0.22 to 0.061, respectively. In order to determine cross-hemispheric coupling changes during epileptiform activity, we subtracted the coherence values of normal activity from those of epileptic traces and considered that any increases equal to or larger than the confidence limit for that trace was significant. For shorter duration aberrant activity (i.e., interictal spikes), we performed a detection analysis based on a threshold amplitude beyond the amplitude distribution of the normal traces (3.5 standard deviations from the mean [29] and computed the average waveform and number of events occurring per unit time).

### Temperature and Activity

We computed temperature averages (F40EET probe) of 5-min periods taken from the hour after the seizure as the difference from the hour prior (e.g., 1 h before seizure average − 5-min temperature average) and statistically analyzed it for comparison. If a seizure occurred in the hour before another seizure, then it was excluded. In the same manner, 1-h activity measures before and following seizure were averaged and compared to assess the impact of seizures on activity (as measured by signal strength changes as the probe moved across the receiver).

### Statistical Analysis

Data are presented as mean ± standard deviation (SD) and were analyzed by repeated measures analysis of variance (ANOVA) and student’s *t* tests (SPSS v.17.0, SPSS Inc., Chicago, IL). The Fisher’s exact test and Mann-Whitney *U* tests were used to compare between models.

## Results

### Baseline EEG in the Collagenase Group

**Fig.2:**
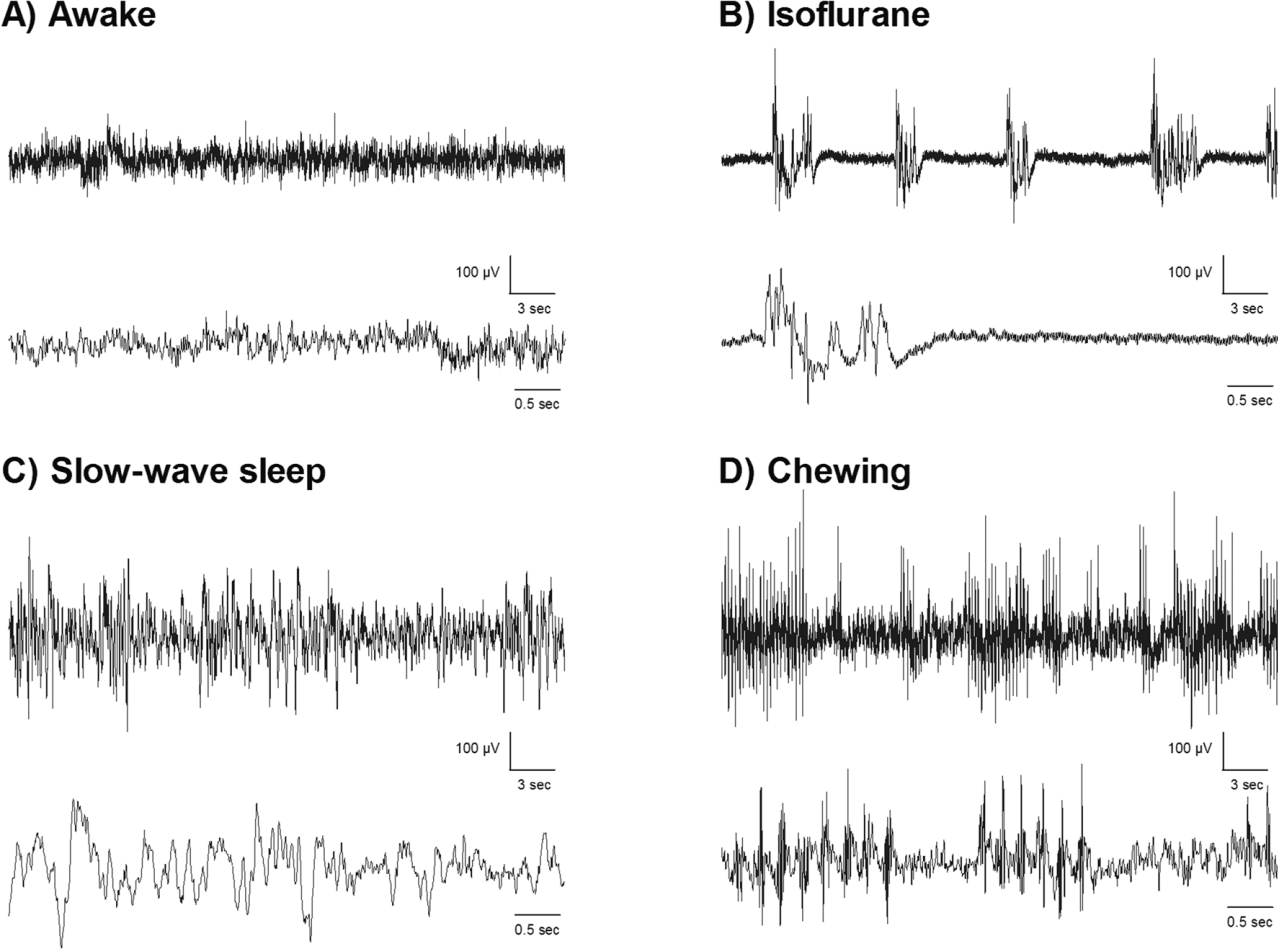
EEG traces during normal behavior. These are examples of EEG activity during **a** awake and alert periods, which display higher frequency and lower amplitude than **c** slow-wave sleep. During anesthetics, such as **b** isoflurane, it is common for EEG to display burst-suppression in rats. We were also able to detect artifacts such as during **d** chewing in the EEG traces. In these instances, the experimenter observed all these behaviors

**Fig.3:**
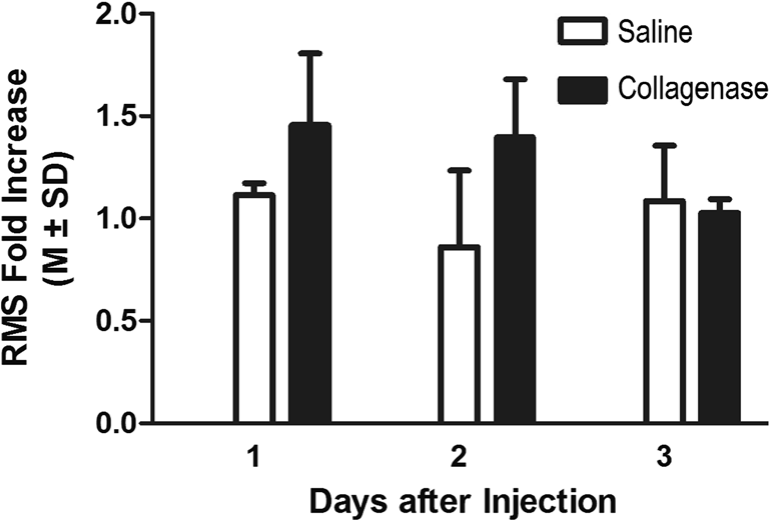
RMS increased in normal EEG after collagenase-induced ICH in the ipsilateral side. An overall RMS-fold increase from the day prior to stroke for a period of 3 days was detected in the collagenase rats that had seizures (*N* = 4) compared to saline injection in the ipsilateral side (*p =* 0.05). This indicates that there were more fluctuations in the EEG traces relating to normal activity after collagenase than after saline (sham surgery) infusion. This was not the case for the contralateral side, for which there was a day effect for both groups but no impact of the treatment

Baseline recordings allowed us to relate different behaviors to the EEG traces (Fig. 2), which were helpful for detecting abnormal activity. When we compared the RMS of 5-min traces of the day prior to stroke/sham surgery and days 1, 2, and 3 after the injection of either saline or collagenase (including only those ICH rats with seizures), we found a group (saline vs. collagenase) effect for the ipsilateral channel (*p* = 0.050, Fig. 3) depicting an increase in RMS in non-epileptic EEG traces after the injection in the collagenase group. This means that the average amplitude fluctuation of traces was higher for the collagenase group even during non-epileptic EEG. Moreover, there was a time effect for the contralateral side (*p =* 0.028), the RMS on the first day after injection was larger than the third day (*p =* 0.040) in both groups, although there was no treatment effect.

### Seizures After Collagenase and Iron Injection

**Fig.4:**
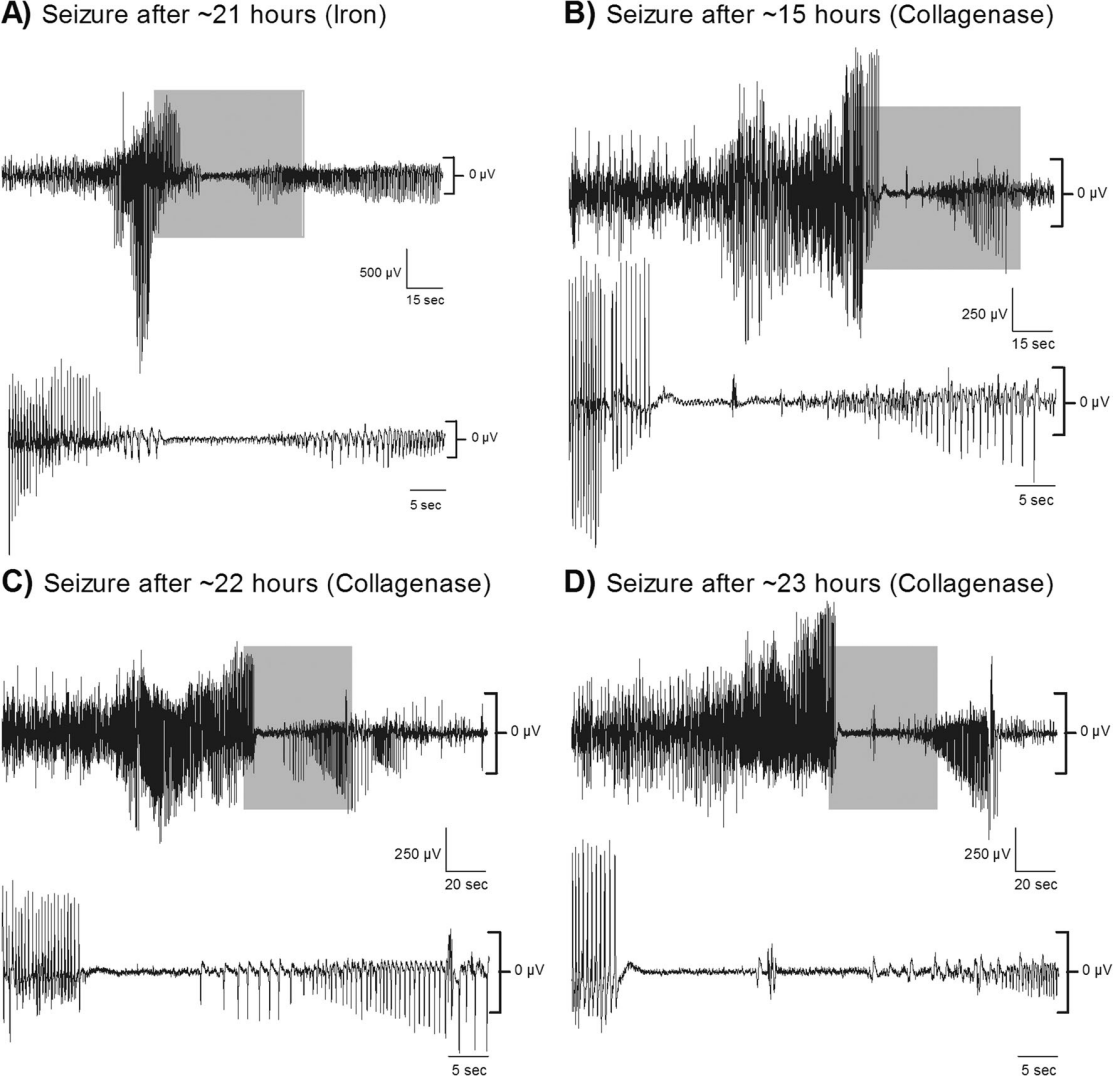
Seizures occurring after iron injection and collagenase-induced ICH. Examples of long-lasting seizures after **a** iron injection as well as **b**–**d** in three different rats given a striatal collagenase-induced ICH. Onset of seizures would be delayed by 10 h or more, and they would range anywhere from 5 to 90 s. Note the characteristic extended periods of suppression (highlighted in *gray* and expanded on the *bottom panels*) after high frequency and amplitude bursting. The confidence intervals (95 %) of normal activity 3 days post-stroke are indicated on the *right of the traces*

One collagenase rat had to be excluded due to equipment failure that did not allow for adequate EEG recordings. Six out of the nine remaining rats (66 %) had seizures within the first 2 days after the stroke with the earliest occurring after ~10 h and the latest occurring after ~36 h. Seizures ranged in duration from ~5 to 90 s, and their averages ranged from 14 to 54 s (Table 1, Fig. 4b–d). Iron also caused seizures within hours of application (Fig. 4a), which displayed a similar pattern of tonic-clonic ictal events followed by notable EEG suppression and occasional afterdischarges. Seizures or other electrographic abnormalities were not observed in any of the six rats infused with 100 μL of autologous blood (*p* = 0.028 for comparing number of rats with seizures between models). Not surprisingly, both the number (*p* = 0.036) and duration of seizures (*p* = 0.036) were significantly greater in the collagenase model.

**Table1:** Characteristics of seizures after collagenase-induced ICH. EEG activity occurring in the first week after collagenase injection were visualized and analyzed. These are the characteristics for the seizures that occurred within the first 36 h after the stroke; no seizures were detected afterwards. For each rat, the number of seizures, laterality, total duration, and time of onset were documented. We also reported other factors to determine the variability of the traces as depicted by the RMS ratio (RMS seizure/RMS non-epileptic activity) and changes in voltage according to their frequency, as depicted by the power increased and frequencies affected. Also, for those frequencies in which the power was affected, coherence was assessed as an indicator of how coupled the activity was in between both channels. Here, coherence was computed as an increase from baseline coherence. Traces shorter than 25 s were not analyzed for power and coherence. Data expressed as *M* ± SD

ID	Incidence	Laterality	Total duration (s)	Time of onset after ICH	RMS ratio	Frequencies affected (Hz)	Power increase (mV^2^)	Coherence change
1	1	Ipsilateral	14	11 h, 52 min	2.44	N/A	N/A	N/A
2	2	Bilateral	102 (51 ± 19.80)	18 h, 50 min	1.9 ± 0.33	↑ 0–4.6	34.11 ± 14.66	−0.53 ± 0.11
3	3	Bilateral	163 (54.33 ± 35.57)	22 h, 7 min	1.92 ± 0.29	↓ 0.5–0.94, ↑1.32–1.5, ↑ 1.78–5.8, ↑12.38–38	16.40 ± 5.23	−0.19 ± 0.10^a^, 0.14 ± 0.16
4	4	Bilateral	108 (27 ± 4.9)	16 h, 25 min	2.65 ± 0.23	↑ 1.07–6.2	16.01 ± 3.99	0.34 ± 0.022
5	8	Bilateral	173 (21.62 ± 17.76)	11 h, 37 min	4.13 ± 0.43	↑ 0–39.9	71.03 ± 14.57	0.42 ± 0.19
6	14	Bilateral	616 (44 ± 46.24)	9 h, 57 min	2.53 ± 0.53	↑ 0–47	33.77 ± 16.07	0.24 ± 0.025

^a^Occurring in frequencies below 5.8 Hz

**Fig.5:**
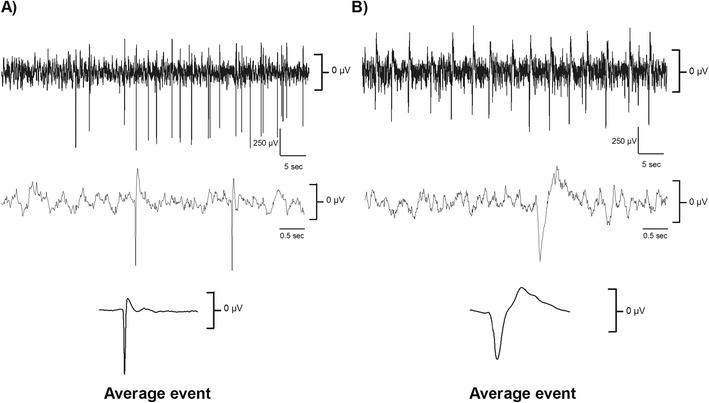
Interictal epileptiform activity in two collagenase rats. In our study, these rats (**a**, **b**) suffered extended periods of aberrant interictal activity. Note the characteristic downward spiking. For rat A, spikes occurred about 20 times per minute, and in rat B, they occurred 10 times per minute. The interictal events elicited over 1 h were averaged, as depicted at the *bottom of the figure*. The confidence intervals (95 %) of normal activity 3 days post-stroke are indicated on the *right of the traces*

In two collagenase rats, we detected extended periods of interictal epileptiform discharges, ranging from ~1.5 to 14 h (Fig. 5). Over the span of 30 min in which seizures occurred in one animal, we detected abnormal interictal events in between obvious ictal activity. Interictal activity did not last more than 2 min; therefore, we were not able to analyze them using our event detection analysis due to their short duration. For the three rats in which 30 days of EEG were screened, two had seizures within 36 h, but none of them had any noticeable EEG abnormalities after that period. Therefore, none of our collagenase rats appeared to have developed epilepsy per se. Most of the seizures were bilateral, which were likely generalized from an initiation zone located in the affected hemisphere and propagated across contralateral hemisphere. There was only one case in which the seizure activity occurred only ipsilaterally (Table 1).

### Severity of Seizures in the Collagenase Group

We calculated RMS and quantified power increases of seizure traces and compared them to non-epileptic activity taken from day 3 post-collagenase surgery as an indicator of increases in amplitude at different frequencies (power) and fluctuations in the amplitude (RMS) of the ictal events. The smallest RMS-fold increase for the ipsilateral side was 1.58 times larger than non-epileptiform activity, and the largest was 4.69 times larger during ictal activity. Similarly, for the contralateral side, the smallest epileptiform RMS was 1.45 times larger, and the largest was 4.96 larger. This indicates that during seizure activity, there was about a 50–500 % increase in amplitude fluctuations in these traces. In general, increases in power were seen at all frequencies up to 38 Hz, with a decrease in frequencies across the 0.5–5.8 Hz bandwidth detected in a single rat (Table 1). Also, in most cases, the ipsilateral channel had an equal or greater increase in power than the contralateral, although this was not the case for two rats. This could support the notion of an ipsilateral focus that propagates, and/or generalizes, to the contralateral hemisphere.

**Fig.6:**
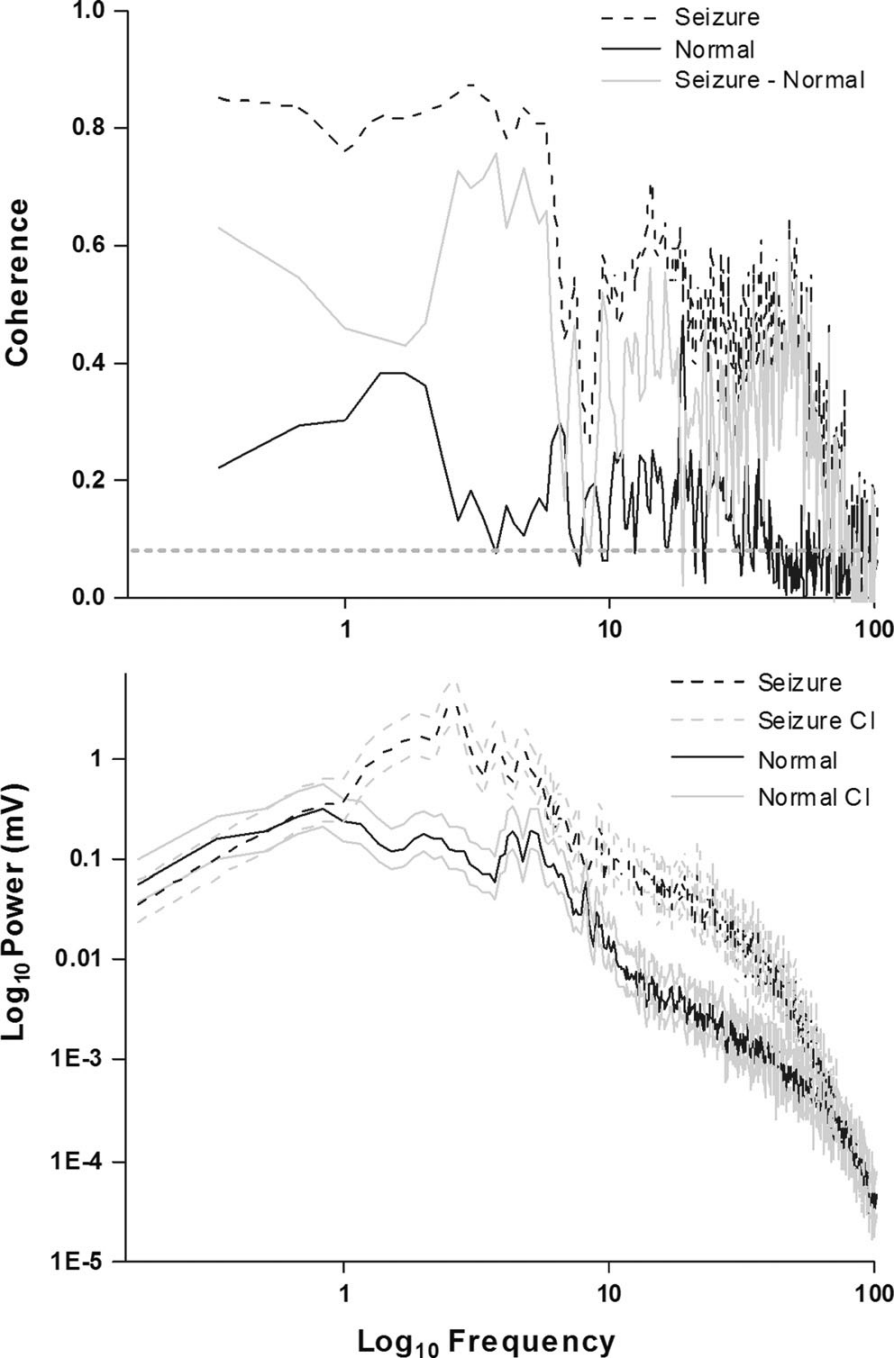
Seizures displayed higher power and increased coherence than normal EEG. This is an example of the coherence (*top panel*) and power spectrum (*bottom panel*) for the epileptiform event in Fig. 4d (*dashed line*) compared to normal EEG activity 3 days after collagenase-induced ICH (*solid line*). Any increase in coherence above the confidence interval limit (*dotted line*) of the difference between seizure and normal activity (*gray solid line*) was significant. The *gray lines* in the power spectrum represent the 95 % confidence interval (CI) and the *black lines* the mean values for the spectrum. For those increased frequencies, there were also increases in coherence

For coherence calculations, we concentrated on increases in coherence at the frequencies in which we noted a change in power. Coherence values range from 0 to 1; values of 0 indicating that signal-specific frequencies between the two channels are completely unrelated, whereas values of 1 indicate that they are completely related. In most instances, we found significant increases in cross-hemispheric coherence during epileptiform activity as compared to normal traces taken 3 days after the collagenase injection (average increase across frequencies 0.28 ± 0.14). Two cases showed exceptions to this rule, one in which no increase was observed and another in which coherence values were significantly decreased despite prominent bilateral seizure activity in both cases. In one rat, we also detected that coherence was decreased for frequencies lower than 6 Hz but that for higher frequencies coherence was increased. Indeed, increases in power for higher frequencies (12–40 Hz) during seizure events were all associated with significantly increased coherence (see example in Fig. 6). This might indicate that the more severe seizures were, the more likely that both hemispheres were engaged in epileptic activity.

### Lesion Volume

**Fig.7:**
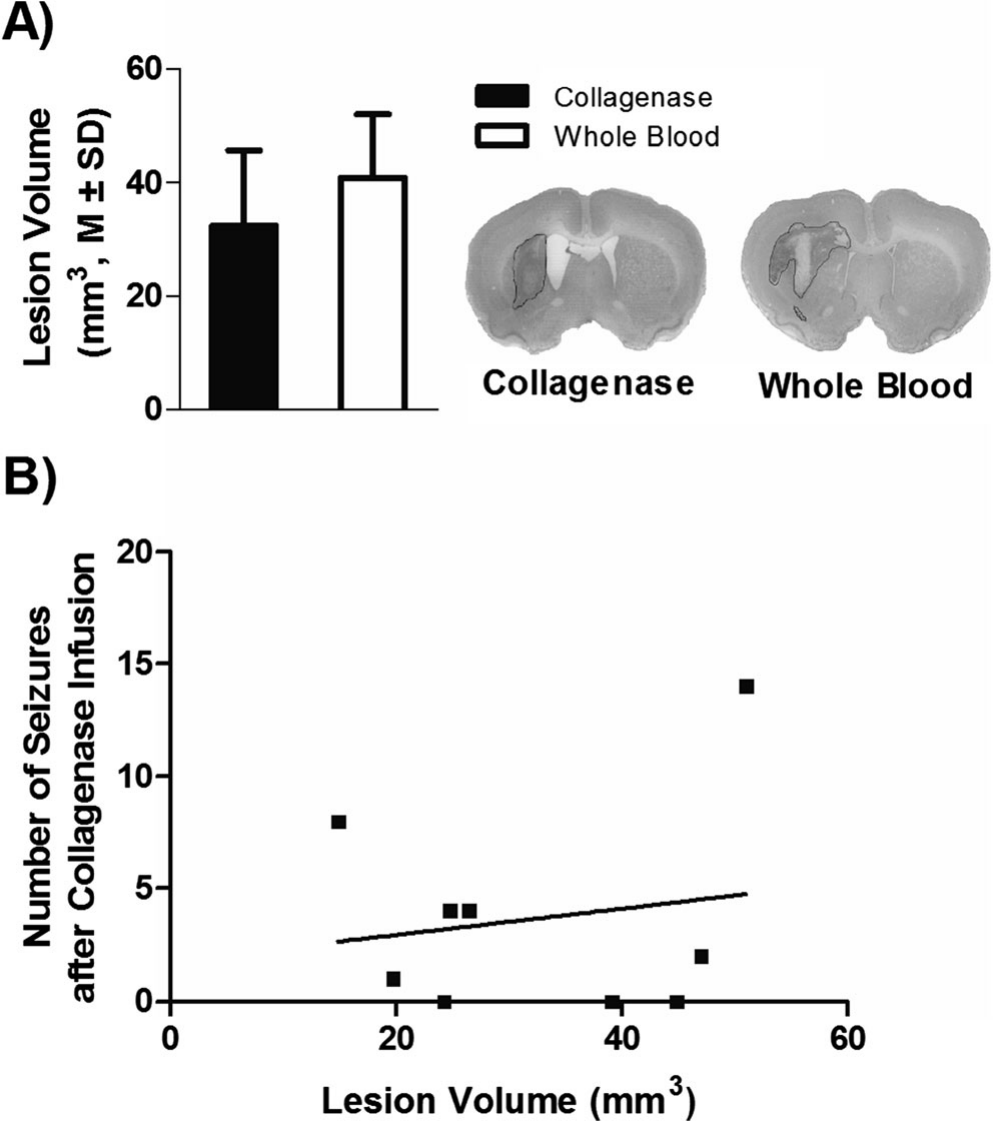
Lesion volume had no relationship with seizure incidence. The ICH typically damaged a substantial portion of the striatum with some damage to corpus callosum. No injury was found in sham-operated rats, other than a needle track. **a** Total lesion volume (*M* ± SD in mm^3^) for the collagenase and whole blood models was not significantly different. Photomicrographs illustrate each model’s profile of injury at the level of maximal damage (cresyl violet stain). **b** There was no significant correlation between lesion volume (*black squares*) and the incidence of seizures in the collagenase model (*r* = 0.16, *p* = 0.67)

The infusion of collagenase or blood caused significant damage and inflammation, as depicted in Fig. 7a (*p* = 0.22 for lesion volume). In the collagenase model, we found no significant relationships between lesion volume and seizure incidence (*r* = 0.18, *p* = 0.65; see Fig. 7b), time of onset after stroke (*r* = 0.30, *p* = 0.56), total (*r* = 0.37, *p* = 0.31) and average time spent in seizure activity (*r* = 0.18, *p* = 0.63), RMS (*r* = 0.18, *p* = 0.65), and power increase (*r* = 0.29, *p* = 0.63). Thus, we did not find that lesion volume predicted seizure characteristics in this model. Owing to the lack of seizure activity, we did not perform this analysis with the whole blood model data.

### Temperature and Activity Data

**Fig.8:**
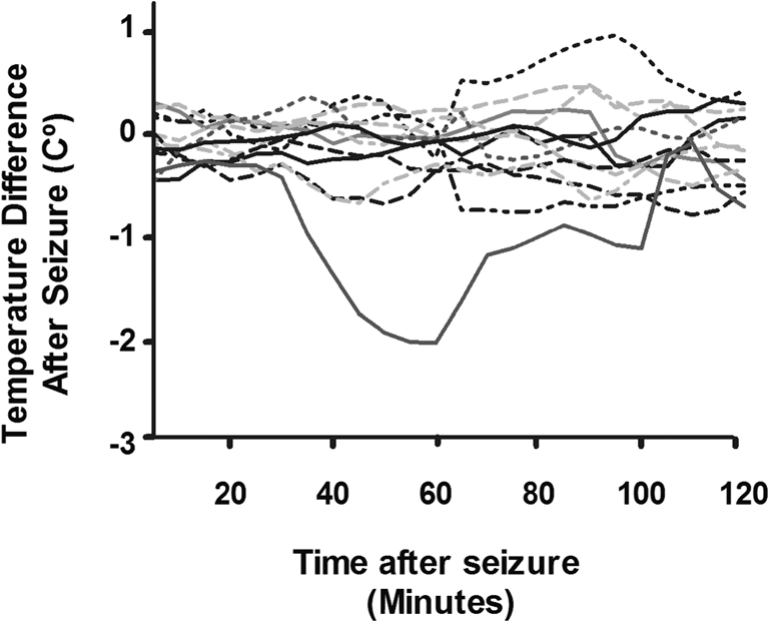
Temperature changes (post-seizure minus pre-seizure values) for 2 h post-seizure activity. Values are expressed as difference between baseline average and temperature (e.g., values below 0 indicate that hypothermia occurred after the seizure). Time 0 would be the time at which rats suffered of a seizure. There were no consistent changes in temperature among the rats

A repeated measures ANOVA did not depict a difference among the twelve 5-min average intervals (i.e., 1 h) following the seizure (*p* = 0.382), indicating that there were no changes in temperature after the seizure. Even though there was no pattern in the temperature change among rats, one rat had a seizure leading to about 90 min of hypothermia (Fig. 8). Moreover, a paired *t* test on all of the rats’ movement activity indicated no difference between activity before and after the seizure (*p* = 0.7968).

## Discussion

We expected seizures to occur in the blood infusion model, but this was not observed. Our results did confirm our hypothesis that seizures commonly occur after striatal ICH in the collagenase model. Sixty-six percent of rats in the collagenase group suffered seizures during the first 36 h following their stroke. Seizures commonly occur after brain injury and stroke, both in patients [30, 2, 31] and in other animal models of brain injury [32–34]. Thus, it is not surprising that collagenase rats would also display abnormal electrical activity as we demonstrated in this study, including both full-blown ictal and abnormal interictal activity, which mostly occurred bilaterally. Increased cross-hemispheric coherence coinciding with increased power suggests that the activity in both hemispheres during seizure activity was coupled and that more severe seizures recruited both hemispheres. Although there were some exceptions to this, coherence remained significantly increased, especially at the higher frequencies. We also demonstrated that even normal-looking electrical activity has an increased RMS for the first 3 days after collagenase-induced ICH, which could indicate that there were abnormalities in non-epileptic EEG activity during this limited time frame. Although robust epileptiform activity was a consistent phenomenon in our collagenase group, we did not find lesion volume to be a predictor for any of the seizure characteristics. Likewise, the lack of seizures in the blood model, which had a comparable lesion, argues against lesion or hematoma volume as key predictors of seizure activity.

The incidence of electrographic seizures in our collagenase group (66 %) is more than double that documented in ICH patients [2]. The difference in incidence might be attributed to the greater range in patient characteristics (e.g., ICH locations, severity) in clinical studies, along with other factors such as species differences. Interestingly, other pre-clinical studies of brain insults, namely, hypoxic-ischemic injury [33], focal ischemia [34], and traumatic brain injury [32], all report a much higher percentage of animals developing seizures and epilepsy than what is reported in the clinic. However, continuous monitoring of EEG for many weeks or months is rare in clinical studies, making it difficult to compare animal and clinical data.

There are key differences between the collagenase and whole blood models that may explain the discrepancy in seizure incidence between these models. For instance, the whole blood model of ICH provides a somewhat different profile of injury (see Fig. 7a) with less secondary injury, inflammation, blood brain barrier damage, and smaller intracranial pressure spikes [23, 35, 36], but these may vary by species [37]. As with inflammation [38], it is possible that the timing, extent, and localization of thrombin production vary between models and this might account for differences in seizure activity. Note that intracerebral infusions of thrombin induce seizure activity [19]. Iron infusions also cause epileptogenic activity [20], as we presently confirmed. However, given the timing of iron release, which in our collagenase model occurs between 24 and 72 h [39], it is unlikely that iron causes seizures as they began between 10 and 22 h, and stopped by 36 h. As well, the hematoma volume is expected to be larger in rats infused with 100 μL of blood than those given collagenase [23]. Thus, if iron were the primary cause of seizures, there should have been more seizures in the whole blood model.

Early seizures are predictors of future epilepsy in stroke patients [13], although a study by Bladin and colleagues [4] showed that all of the patients that had late onset seizures, which were those at 2 or more weeks after the stroke, developed epilepsy. We did not find recurrent seizures after the first 36 h of the stroke, even though we screened EEG for up to a month after collagenase infusion. While this suggests that this model does not lead to epilepsy, a much larger sample size is needed especially given the small percentage expected to develop that condition. There is also the possibility that seizures may develop later than a month after ICH in rats, as occurs in other animal models such as traumatic brain injury [32].

There are some limitations to this study. First, we did not video record any of the seizure events, so the type of behavioral manifestations with these electrographic seizures remains unknown. Although, we occasionally noticed behavioral signs of focal seizures, such as clonic paw movements. Second, the relatively limited number of animals in this study cannot exclude the possibility that occasional seizure activity occurs in the whole blood model. Third, with larger sample sizes, a modest relationship between lesion volume and seizure characteristics may have been detected. Indeed, others have reported a relationship between epileptiform activity and infarct size after focal ischemia [40]. In a clinical study, however, small lesion size was a better predictor of seizure incidence [3]. Fourth, while we recommend use of telemetry probes, tethered systems have the advantage of allowing monitoring from more locations, which would be advantageous in future studies (e.g., to identify seizure focus). The use of telemetry probes also had some additional disadvantages (e.g., greater cost) including technical problems we encountered with the use of lead extenders and of course the inevitable loss of battery power. Lastly, while EEG eventually returned to normal, it is likely that seizure thresholds were altered as found in traumatic brain injured rats given a pro-convulsant challenge [32].

Further studies should be carried out to advance our knowledge of seizures occurring after ICH. Seizure incidence should be studied after changing the location of the lesion, as lobar/cortical location has been associated with more seizure activity in patients [3, 4]. Even though the striatal model of ICH is a common one, other structures have also been targeted, such as cortex [18] and hippocampus [41], and we are presently evaluating these models. It is possible that whole blood injections in different locations may elicit seizure activity. Also, patients with an ICH also have increased ICP after the insult [15], which is also common after a collagenase-induced ICH in rats [36]. This sustained rise in ICP could be associated either to the mass effect arising from the hematoma and edema or to seizure activity, which could especially be related to ICP spiking [9]. This could be elucidated by simultaneous EEG and ICP monitoring. Furthermore, future research should focus on the relationship between seizures, cell death, and recovery. Some clinical studies have related seizures with worsened outcome and mortality [12–14], although others failed to do so [11, 2–4]. In animal models, we can experimentally increase seizure activity with convulsant drugs or diminish it with AEDs and test its impact on several markers of cell death (e.g., neurodegeneration) and functional outcome.

In conclusion, seizures occur in the majority of rats subjected to a collagenase-induced striatal ICH but did not occur after infusion of whole blood—models widely used to study the pathophysiology of ICH and to assess neuroprotectants and rehabilitation therapies [35]. As ICH patients also suffer from seizures early after the stroke, the rat collagenase model has good face validity to model seizures occurring after ICH, although further work is needed to determine whether the underlying cause is the same as in patients. This is a key factor for translational purposes, as others have raised concerns regarding differences between animal and human ICH pathophysiology [42, 43, 35, 44–46]. Researchers should also consider that seizures could potentially impact their studies. For instance, seizure activity may exacerbate the damage caused by the stroke, altering the effectiveness of neuroprotective therapies. Also, treatments may indirectly reduce cell death by ameliorating seizure activity. We recommend the use of the whole blood model when seizures may be a confounding factor. As this is the first study to find that seizures occur after collagenase-induced ICH in rats, we encourage further research to understand the relationship between seizures, cell death, and recovery after ICH. This way, we will be able to enhance therapies currently provided to ICH patients.
